# Providing Services During Times of Change: Can Employees Maintain Their Levels of Empowerment, Work Engagement and Service Quality Through a Job Crafting Intervention?

**DOI:** 10.3389/fpsyg.2020.00087

**Published:** 2020-01-28

**Authors:** Inge L. Hulshof, Evangelia Demerouti, Pascale M. Le Blanc

**Affiliations:** Human Performance Management Group, Eindhoven University of Technology, Eindhoven, Netherlands

**Keywords:** customer ratings, empowerment, job crafting intervention, organizational change, service quality, work engagement

## Abstract

By means of a quasi-experimental study, the effects of a tailor-made job crafting intervention for employees of a Dutch unemployment agency were evaluated. The intervention was designed to prevent a decrease in employee empowerment, work engagement and employee performance (i.e., the provision of services) due to organizational changes. Seventy-four employees received a 1-day training in which they set four job crafting goals for the subsequent weeks. After 6 weeks a reflection session was organized. Repeated measures ANOVA’s showed that the intervention prevented a decrease in employees’ feelings of empowerment. Furthermore, pre-post comparison tests showed that the control group (*N* = 89) experienced a significant decrease in work engagement, whereas the intervention group did not. Results showed no effect on customer-rated employee service quality. However, 1 year after the intervention, customer ratings of employee service quality were significantly higher for the intervention group compared to the control group. Although further research is needed, our results demonstrate that a job crafting intervention may be a promising tool to combat a decline in employee empowerment and work engagement during times of organizational change.

## Introduction

The nature of work has undergone some major changes in the last couple of decades. It has, for example, become more service-oriented (Dall’erba et al., 2009) influencing the type of performance that employees need to deliver. In the past, employee performance mainly referred to the number of products made, whereas nowadays it has gradually changed toward the quality of services provided (Oliva and Sterman, 2001). Employee performance is not the only thing that has changed: new technologies, economic forces, social innovations, new ways of working, they all force employees to adapt at a high pace (Kompier, 2006). Organizations have to innovate, change and improve themselves time after time to stay ahead of the competition and to keep their customers satisfied. However, adapting to a changing environment is not always easy. Employees may be insecure or even cynical about upcoming changes, which may in turn – inadvertently – affect their performance (Cartwright and Holmes, 2006). To prevent this from happening, it is extremely important to examine ways in which employees can adapt to changes in a positive way. In this article we focus on a promising bottom–up approach through which employees can make small changes in their work to align it more with their personal wishes and preferences: job crafting (Wrzesniewski and Dutton, 2001). Job crafting is defined as “the physical and cognitive changes individuals make in the task or relational boundaries of their work” (p. 179). It describes how employees proactively shape their work context by changing (a) the type and number of tasks they carry out, (b) the way they interact with others at work and (c) the way they think about their work (Wrzesniewski and Dutton, 2001). In the present study, we will explore the effects of a job crafting intervention during times of organizational change. The intervention focuses on increasing job crafting behavior, in order to prevent a decrease in work engagement, empowerment, and the provision of high-quality services.

This study contributes to the literature in several ways. First, it was conducted during times of organizational change. Employees nowadays have to adapt to many changes (e.g., innovations, advanced technologies, new ways of working) as our world is ever-changing (Van den Heuvel et al., 2010). Adapting to these changes may be challenging, as it can cause stress, increase insecurity and reduce motivation, all potentially undermining employee performance (Callan, 1993; Caldwell et al., 2004; Gilley et al., 2009). Therefore, it seems worthwhile to not only examine the effects of interventions during ‘quiet’ times, but also explore their effects during times of change to see if they enable employees to adapt more easily (Demerouti et al., 2017). Second, as providing high quality services is a key performance indicator in the service sector (Bowden, 2009), developing and validating interventions that stimulate this is extremely valuable. The current intervention does so by focusing on enhancing service-oriented task performance (i.e., performing one’s service-related tasks optimally) and empowering services (i.e., providing services that enhance customers to feel confident to take care of their own (work-related) affairs). Although the current study focuses on the unemployment sector, the results are valuable for other service organizations as well, since both components of performance/service quality focus on helping customers in the best way possible. Third, by taking customer satisfaction into account, this study uses a unique research design. We do not only rely on self-report measures of service quality, but also examine whether the effects of the intervention are noticeable for others (Gordon et al., 2018). As customer satisfaction is of vital importance for service-organizations (Taylor and Pandza, 2003), the current job crafting intervention aims to directly contribute to this key performance indicator in the sector too. Lastly, although job crafting has limitedly been investigated in the service sector (e.g., McCelland et al., 2014; Hulshof et al., 2019), we are unaware of any job crafting intervention studies in this sector. As more and more organizations nowadays are providing services (Dall’erba et al., 2009), examining the effects of job crafting in this sector is not only of theoretical value (broadening its generalizability) but also of great practical value.

### Job Crafting and the Job Crafting Intervention

Job crafting has been framed within the Job Demands-Resources (JD-R) model (Tims and Bakker, 2010). This model describes the relationship between work characteristics and employee well-being (Demerouti et al., 2001) and proposes that each work environment has its own unique configuration of job demands and job resources (Bakker and Demerouti, 2017). Job demands refer to those aspects of the work (either social, psychological, physical or organizational) that require sustained physical or psychological (emotional or cognitive) effort. Job resources are those aspects of the work (either social, psychological, physical or organizational) that help achieve work related goals, reduce the effects of job demands and stimulate personal growth, learning and development (Demerouti et al., 2001). Although all job demands require the investment of effort and are associated with certain costs, we distinguish between two types of job demands: hindering job demands and challenging job demands (Crawford et al., 2010). Hindering demands refer to demands that ‘involve excessive or undesirable constraints that interfere with or hinder an individual’s ability to achieve valued goals’ (Cavanaugh et al., 2000, p. 67). Challenging demands are those demands that may cause a stress response while pursuing, but in the end are seen as rewarding and worth the effort (Cavanaugh et al., 2000).

When crafting their job, employees can make changes to their job demands and job resources using three strategies: (1) decreasing hindering demands (e.g., making the work emotionally less intense or avoiding making difficult decisions), (2) seeking challenging demands (e.g., starting a new project or following a course on a topic of interest) and (3) seeking resources (e.g., asking for feedback or increasing the variety of tasks) (Petrou et al., 2012). Some researchers (e.g., Tims et al., 2012) further specified seeking resources into two categories: social resources and structural resources. Social resources are resources related to the social aspects of the job (e.g., support and feedback) while structural resources are resources that are related to the job design (e.g., autonomy and opportunities for development). In the present study, we will make this distinction too.

A job crafting intervention may be beneficial, especially during times of change, as job crafting enhances the person – environment (P-E) fit (Tims et al., 2012). Due to top–down organizational changes, the P-E fit of employees is shifting, possibly leading to a less optimal fit and consequently performance (Caldwell et al., 2004). Providing people with tools (i.e., job crafting strategies) to – at least to some extent – restore their P-E fit may be a valuable bottom–up approach for employees to deal with their changing environment. Training people to craft their job may enhance feelings of control, as job crafting behaviors are unsupervised, voluntary and beneficial for the employee (Wrzesniewski and Dutton, 2001). Thus, although people cannot prevent the top–down organizational changes from happening, they may, through job crafting, expand strategies that are needed to stay in control and to adapt to the changes.

Previous job crafting interventions have provided valuable results in different organizational settings. For example, Van den Heuvel et al. (2015) showed that a job crafting intervention is a promising means to enhance personal resources (i.e., self-efficacy) and well-being for police officers. Moreover, Gordon et al. (2018) showed that a job crafting intervention enhances employee performance in healthcare, whereas Dubbelt et al. (2019) showed that a job crafting intervention, executed in an educational setting, was able to enhance employee work engagement. Lastly, Demerouti et al. (2017) showed that the intervention was effective to increase positive affect and openness to change among municipality employees during organizational change due to austerity. In general, results suggest that the job crafting intervention is effective in stimulating aspects of job crafting behavior, although the effects cannot always be detected with multivariate tests (Demerouti et al., 2019). Demerouti et al. (2019) suggested that it might be difficult to detect the effect of the intervention on job crafting behaviors because the scale may not include the whole range crafting behavior or there might be a ceiling effect of behaviors that individuals are involved prior to the intervention such as seeking resources. However, a recent meta-analysis of 14 job crafting interventions concluded that the intervention has significant on overall job crafting but also on seeking challenges and somewhat stronger on reducing demands (Oprea et al., 2019).

Our intervention is in line with earlier job crafting interventions and is designed based on experiential learning theory (Kolb and Kolb, 2011). Experiential learning theory emphasizes the importance of past experiences in learning and behavioral change. All four stages relevant for learning to apply job crafting techniques and to initiate actual behavioral change were incorporated in the intervention (for an overview of the intervention see Tables 1A,B). The learning process begins with concrete experiences with the behavior, followed by reflection (stage 1 and 2) (Kolb et al., 2000). In the third stage, individuals have abstract ideas about the new behavior and how to benefit from it (Kolb and Kolb, 2011). In this stage it is important to emphasize the added value of the new behavior to enhance individual’s motivation to invest time and energy trying to implement it. In the fourth and last stage, employees actively experiment with the behavior to create new experiences (Kolb et al., 2000). In order to stimulate the implementation of the newly learned behavior, goal setting is extremely important (Schunk, 1990), so we explicitly focused on that too during the intervention. As learning is an ongoing process, after stage four, individuals start again in stage 1 (Kolb et al., 2000; Kolb and Kolb, 2011). Thus, building on the experiential learning theory we expect that our job crafting intervention will stimulate employees in the intervention group to learn to apply and integrate job crafting techniques into their work routines.

**TABLE 1A:** Overview of the intervention at day 1.

Time spent*	Steps	Aspects of the intervention reflecting experiential learning theory
15 min	1: Concrete experiences	• Providing real-life examples of job crafting and empowering service, based on interviews conducted with employees of the unemployment agency.
85 min	2: Reflection	• Mapping exercise (Davies, 2011) in which participants mapped a normal work week and distinguished between energizing aspects (resources) and aspects that cost energy (demands) based on the JD-R model (Bakker and Demerouti, 2017). • Learning by analogy exercise (Carbonell, 1983) in which participants retrieved past (successful) experiences with job crafting and empowering service and reflected (in small groups) upon the value of these experiences for current situations.
40 min	3: Abstract concepts	• Using the JD-R model (Bakker and Demerouti, 2017), explaining participants how job crafting benefits work-related outcomes, such as performance (Bakker et al., 2012) and work engagement (Demerouti et al., 2015). Explaining the concept of empowering service.
95 min	4: Creating new experiences	• Setting 4 SMART (specific, measurable, attainable, realistic and time-bound) goals (Doran, 1981) for the weeks after the first day of the intervention (i.e., week 1: seeking (social) resources, week 2: reducing demands, week 3: seeking challenges, week 4: seeking (structural) resources). By setting these goals, participants could practice with all job crafting strategies at least once. • In couples: thinking about possible facilitating factors and obstacles for the goals that were set. This helped participants explore possible obstacles (how to deal with them) and facilitating factors (how to optimally use them) in reaching their goals. • Weekly reminders (per e-mail) were sent to encourage goal achievement (Fry and Neff, 2009)

*The overall amount of time does not add up to the total duration of the intervention (5.5 h; 330 min), as introduction (25 min), closure (10 min), and breaks (60 min) were included in the total duration of the intervention, but are excluded in this overview as they do not resemble a specific element of experiential learning theory.

**TABLE 1B:** Overview of the intervention at day 2 (evaluation session).

Time spent*	Steps	Aspects of the intervention reflecting experiential learning theory
25 min	1: Concrete experiences	• The real-life job crafting goals and experiences in the weeks between day 1 and 2.
45 min	2: Reflection	• Celebrating successes to enhance ownership and self-confidence (e.g., Sawyer et al., 2007) • In small groups: reflecting upon the goals set in the weeks between day 1 and 2. Finding obstacles, discussing how to deal with them and explore ways in which facilitating factors can be used to reach the desired goal(s).
10 min	3: Abstract concepts	• Emphasizing again the benefits of job crafting based upon the JD-R model (Bakker and Demerouti, 2017)
15 min	4: Creating new experiences	• Looking ahead: discussing with participants how to implement the use of job crafting strategies into their (daily) work routines.

*The overall amount of time does not add up to the total duration of the evaluation session (2 h; 120 min) as introduction (5 min), closure (10 min), and breaks (10 min) were included in the total duration of the intervention, but are excluded in this overview as they do not resemble a specific element of experiential learning theory.

H1: Employees participating in the job crafting intervention will show increased levels of (a) increasing structural resources, (b) increasing social resources, (c) decreasing hindering demands and (d) increasing challenging demands after the intervention compared to employees in the control group.

### Well-Being During Times of Change

Providing high quality services is a key performance indicator in the service sector, regardless of organizational changes going on. Therefore, finding ways to stimulate this type of performance seems worthwhile. In our intervention we did so by focusing on work engagement and empowerment, both factors that are related to intrinsic motivation (Thomas and Velthouse, 1990; Bakker and Demerouti, 2009). Intrinsically motivated people are willing to pursue time and energy into the tasks at hand, leading to higher levels of performance. When people are engaged in their work, they are enthusiastic and feel energized while working (Schaufeli et al., 2002). When empowered, employees feel they are able to proactively shape their work role and context in order to carry out their work (Spreitzer, 1995). These factors differ in that work engagement is related to interest and excitement, whereas empowerment is related to confidence.

Work engagement consists of three dimensions: *vigor, dedication* and *absorption*. When vigorous, people feel energized by their work and are resilient during setbacks. When dedicated, people are enthusiastic and continue until the job is done. When absorbed, people are highly focused and lose track of time (Schaufeli et al., 2002). Work engagement has extensively been linked to performance (for a review, see Christian et al., 2011), in that employees who are more engaged, have the energy and the willingness to devote their attention to their tasks and perform them better. Recent research has shown that job crafting seems a promising tool to enhance work engagement even during organizational change (e.g., Petrou et al., 2018). Employees who proactively craft their job, experience an increased fit between themselves and their work (Bakker et al., 2012) by focusing on those aspects of the work that are significant and important to them (Pratt and Ashforth, 2003), resulting in higher levels of energy and enthusiasm about their work; they become more engaged. Engagement may be especially beneficial during times of change, as engaged employees are more creative and willing to go the extra mile (Van den Heuvel, 2013). As organizational change is hardly ever a smooth process (Cartwright and Holmes, 2006; Kompier, 2006), creativity and extra effort may be extremely valuable to be able to handle unforeseen, difficult or challenging situations (Petrou et al., 2018). Furthermore, work engagement is contagious (Bakker and Demerouti, 2009), which implies that as one person is engaged, his/her engagement may crossover to another person. This crossover effect may be especially valuable in times of change. Work engagement, as a counterforce to possible cynicism regarding the organizational changes, may help employees adapt to changes in a positive way. Moreover, this positive adaptation may crossover to others, amplifying its effects (Van den Heuvel et al., 2010).

Empowerment refers to increased intrinsic task motivation through delegation of responsibilities and authority to the lowest organizational level possible (Thomas and Velthouse, 1990). There are four underlying mechanisms to empowerment: *meaning*, *impact*, *competence*, and *self-determination*. Meaning is described as the value an individual gives to a task goal or purpose, based on the individual’s ideals and standards (Thomas and Velthouse, 1990). Impact is described as the influence an individual has to control strategic, operational or administrative organizational outcomes (Ashforth, 1989). Competence (or self-efficacy) is described as the belief an individual has to be capable to complete the tasks at hand (Gist, 1987). Self-determination is described as the sense of having a choice to initiate and regulate behaviors, for example making decisions about work pace and the order in which tasks are carried out (Spreitzer, 1995). Thus, when people get adequate responsibilities and authority in their work, they can feel empowered and are intrinsically motivated to carry out their work-related tasks through a sense of meaning, impact, competence and self-determination. Empowerment has been linked to performance (e.g., Chen et al., 2007; Seibert et al., 2011), in that people who feel more empowered, perform better. Empowerment may also boost customer satisfaction, as empowered employees have the confidence to handle customer needs and problems efficiently (Chiang and Jang, 2008). Since the service sector is people-oriented in essence, empowerment, especially during times of change, may be a promising ‘tool’ to enhance the sector’s key performance indicator: the provision of services. We expect that empowerment can be stimulated through the job crafting intervention. When people craft their job, they enhance the fit between themselves and their job (Bakker et al., 2012) by focusing on those aspects of the work that are significant and important to them (Pratt and Ashforth, 2003). This results in higher levels of intrinsic (task) motivation (Ryan and Deci, 2000) through a sense of meaning, impact, competence and self-determination. That is, they become empowered. Empowering employees during organizational change may be beneficial, as it helps employees feel less powerless (Conger and Kanungo, 1988) when adapting to the organizational changes. This way, they can maintain their service quality level.

Organizational change may negatively impact employee health and well-being, as it causes stress, anxiety and ambiguity (Callan, 1993; Cartwright and Holmes, 2006). Having to deal with the additional demands from the implemented changes and providing employees with insufficient resources, may negatively affect their work engagement and empowerment (Lamm and Gordon, 2010; Bakker and Demerouti, 2017). In our study population, management decided to double all targets, leaving employees with twice as many clients to provide services to. Moreover, employees did not get additional resources (personnel, time, etc.). Therefore, we expected employee well-being to decline. However, employees may be able to temper this process through job crafting, as organizational change from a JD-R perspective refers to a shift in job demands and job resources to which employees have to adapt (Van den Heuvel et al., 2010). Job crafting is targeted at maximizing job resources and challenges and minimizing hindering job demands. Thus, as a means to restore the configuration of job demands and job resources, job crafting may help to uphold work engagement and empowerment during times of change (Miller, 2015; Petrou et al., 2016, 2018). Therefore, we expect that during times of change employees participating in the job crafting intervention will be able to maintain their levels of work engagement and empowerment, whereas employees in the control group will experience a decrease as a result of the heightened demands.

H2: Employees in the control group will experience a decrease in (a) work engagement and (b) empowerment, whereas employees in the intervention group will not.

### Employee Service Quality in Times of Change

In a people- or customer-oriented sector like the service sector, providing high quality services is of vital importance (Chiang and Jang, 2008). Therefore, our intervention will focus on two types of service quality: service-oriented task performance and empowering service. Service-oriented task performance, which finds its roots in task performance (Motowidlo and Van Scotter, 1994), is defined as ‘the provision of high quality services to customers and clients in order to meet organizational and customer goals’ (The authors, submitted). The construct highlights the importance of the quality of the relationship between client and service providing employee (Oliva and Sterman, 2001) through active listening, expectation management and emotional support. When applying service-oriented task performance to the unemployment sector, it refers to the providing of high-quality services to unemployed candidates and potential employers to effectuate successful mediation between the unemployed candidate and his/her potential new employer.

Empowering service finds its roots in the concept of empowering leadership. Empowering leadership involves a replacement of power from management to employees who have the autonomy and the capacities to take initiative and make decisions about daily events (Amundsen and Martinsen, 2014). The leader-subordinate relationship is hierarchical, just as the relationship between service employee and unemployed customer. Service employees not only support their customer, they are also allowed to sanction them when they do not follow the rules regarding their unemployment benefits. Thus, as both the leader-subordinate and the service employee-customer relationship are hierarchical, empowering leadership may be applicable in the service context too. We labeled this type of service ‘empowering service’ and it refers to the replacement of power from service employee to unemployed customers who have the autonomy and capacities to take initiative and feel responsible to make decisions about their (daily) job seeking process.

We expect employees participating in the job crafting intervention to be able to maintain their levels of service-oriented task performance and empowering service during times of organizational change. When crafting their job, employees can shape the conditions (resources and challenges) necessary to perform optimally. At the same time, job crafting can help diminish the effect of hindering or stressful work aspects that interfere with optimal performance (Tims and Bakker, 2010). Both aspects seem especially valuable during times of change, as top–down changes can cause cynicism and resistance, potentially undermining employee performance (Caldwell et al., 2004; Demerouti et al., 2017). If employees, undergoing organizational change, are able to mobilize their resources and diminish the effects of the hindering work aspects related to the organizational changes going on, they may be able to maintain their levels of performance (Van den Heuvel, 2013). When employees craft their job, they are able to create a more satisfying work context for themselves, enabling themselves to maintain to provide high quality services (i.e., service-oriented task performance and empowering service). Thus, as organizational changes are being implemented, we expect employees in the job crafting intervention to maintain their levels of service-oriented task performance and empowering service, while employees in the control group will show reduced levels of service-oriented task performance and empowering service.

H3: Employees participating in the job crafting intervention will be able to maintain their levels of (a) service-oriented task performance and (b) empowering service while employees in the control group will show a decrease.

Finally, we aimed to examine whether the intervention has effects that are observable for others as well. Therefore, this study also takes customer-rated performance measures into account. We examine, 5 months and 1 year after the intervention, whether customers of employees in the intervention group rate the performance of their advisor more positively than customers of employees in the control group. The timeframe of 5 months is based on the work of Lally et al. (2010), who showed that it takes about 66 days for habits to form. As employees do not work 7 days a week, or even work full-time, we collected customer rated performance measures 5 months after the intervention, making sure all employees had at least 66 days to practice the newly learned job crafting techniques. Moreover, we collected customer rated performance measures after 1 year, as by then, the organizational changes were fully implemented. We examine whether the effects of the intervention were still noticeable for customers during ‘quiet times.’

H4: (a) Five months and (b) one year after the intervention, customer-rated service quality (i.e., service-oriented task performance and empowering service) will be higher for employees participating in the intervention compared to employees in the control group.

**FIGURE 1:**
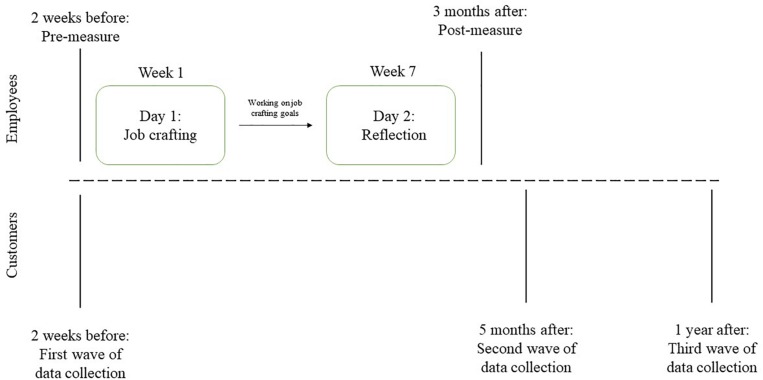
Overview and timeline of the intervention.

For a full overview of the intervention and its timeline, please see Figure 1.

## Materials and Methods

### Study Design

The intervention took place in a department of a Dutch unemployment agency. This department consisted out of three separate buildings. For the intervention two buildings were selected (based upon their size) as the intervention group. The other one served as the control group. People were recruited by posting small messages in the weekly newsletter and by giving more detailed presentations during work meetings. As the department shared the same newsletter, we could not prevent that people in the control group knew they were in the control group. However, the control group did not receive the more detailed presentations in their work meetings. As both groups worked in different buildings and had little contact with each other, the cross-over effects from the intervention to the control group were kept as limited as possible. Participation in both groups was voluntary and participants could drop out at any moment. Overall, nine workshops, with a maximum of 12 participants per group were given. Maximum group size was determined upon previous job crafting interventions (e.g., Van den Heuvel et al., 2015). The workshops were given at an external training facility. After the study was completed, people in the control group were offered to participate in the training as well, such that they could learn how to implement job crafting into their work routines too. All employees of the department of the Dutch unemployment agency in which the intervention took place were invited for a debriefing session in which the results of the intervention were discussed. Moreover, results were shared via the weekly newsletter.

The Dutch unemployment agency is a politically oriented organization, as their policies come from several Dutch ministries and depend upon the political landscape that regularly (mostly once every 4 years) changes. During the intervention, no political changes were affecting the organization, making it a ‘quiet time’ in the organization. However, local departments have some freedom to operationalize their work processes. Management in the departments participating in the intervention decided to double all targets. This meant that service employees had to have twice as many counseling sessions with their unemployed customers. Moreover, the workload of the supportive staff increased as the administrational tasks intensified. Thus, without getting additional capacity, employees had to work up to twice as efficient as before.

### Participants

**FIGURE 2:**
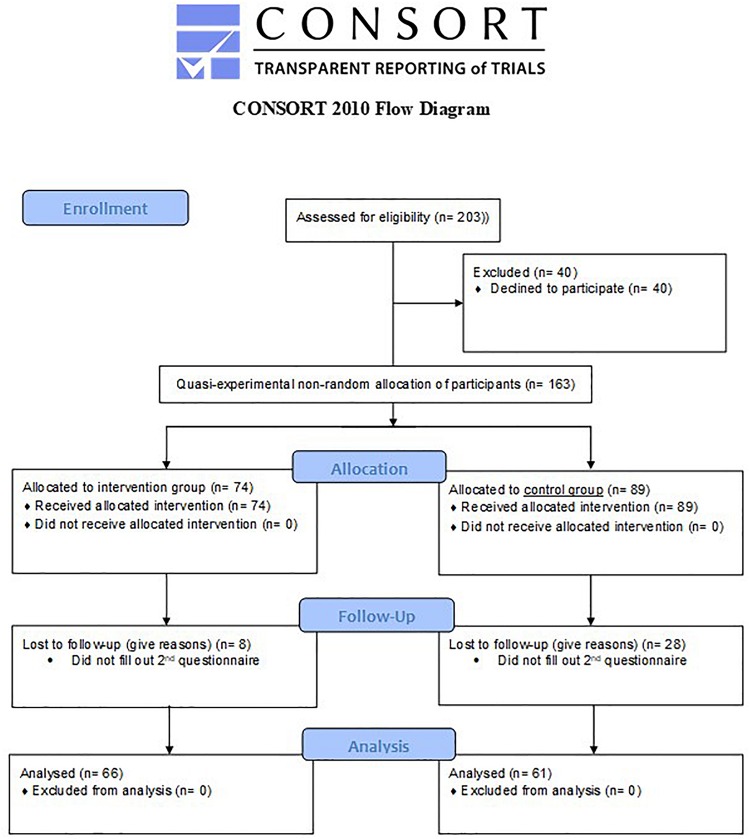
CONSORT Flow Diagram (Moher et al., 2001).

At T1 the intervention group consisted of 74 employees and the control group of 89 employees. The intervention group consisted of 49 women (66.2%) and 25 men (33.8%). The dropout at T2 was 13.2% in the intervention group and 31.5% in the control group, leaving *N* = 66 for the intervention group and *N* = 61 for the control group respectively (for an overview please see the CONSORT Flow Diagram, Figure 2). The dropout pattern was completely random (*MAR/MCAR*; χ^2^ = 128.3, *df* = 133, *p* = 0.60) and participants who dropped out at T2 did not significantly differ from the other participants at T1 on the study variables (i.e., job crafting, work engagement, empowerment, empowering service and service-oriented task performance). Two weeks before the start of the intervention, both the intervention- and the control group were asked to fill out a pre-intervention questionnaire. Three months after the intervention, they were asked to fill out a post-intervention questionnaire. On average participants in the intervention group were 46.1 years old (*SD* = 10.2), worked 18.1 years (*SD* = 11.3) for the current organization, worked 5.3 years (*SD* = 6.0) in their current position and worked 32.6 h (*SD* = 6.6) a week. The control group consisted of 60 women (67.4%) and 29 men (32.6%). On average participants were 46.3 years old (*SD* = 10.9), worked 18.23 years (*SD* = 11.2) for the current organization, worked 5.2 years (*SD* = 5.0) in their current position and worked 31.5 h (*SD* = 7.2) a week. The control group matched the intervention group based on gender, age, tenure and position in the organization. Please see Table 2 for an overview of all biographical data, specified per time point.

**TABLE 2:** Biographical information of all participants at T1 and T2.

Biographical information	Intervention group		Control group	
	T1 (*N* = 74)	T2 (*N* = 66)	T1 (*N* = 89)	T2 (*N* = 61)
(1) Gender	Male: 25 Female: 49	Male: 24 Female: 42	Male: 29 Female: 60	Male: 22 Female: 39
(2) Age	46.1	45.9	46.3	46.5
(3) Tenure	18.1	18.0	18.2	18.8
(4) Current position	5.3	5.6	5.2	5.0
(5) Working hours per week	32.6	32.8	31.5	32.1
(6) Dropout percentage	–	13.2%	–	31.5%

### Customer Ratings

Two weeks before the start of the intervention, 5 months afterward and 1 year after the intervention we collected customer ratings data to explore whether the effect of the intervention on stimulating the providing of high-quality services was also noticed by unemployed customers. Before the start of the intervention, 96 responses (34 intervention group; 62 control group) were collected. After 5 months 104 responses (34 intervention group; 69 control group) were collected. One case provided no details about the advisor. Therefore, this case was excluded from further analyses. After 1 year 201 responses (68 intervention, 131 control group) were collected. Again, one case lacked details about the advisor. Therefore, this case was excluded from further analyses. Recruitment of participants was done on site several days. The first author asked participants who happened to have an appointment with one of the advisors of the Dutch unemployment agency that day whether they were willing to fill out a short form after their appointment. The first author sat at the different departments for several days during each time point, to make sure that as many advisors as possible were included in the responses. If (at all three measurement points) there was more than one assessment of a specific advisor, the average of these assessments was used. This resulted in one (averaged) score per advisor per time point. There were some missing values for some of the customer assessments (as not all aspects of empowering service may occur during each appointment an advisor has). Therefore, we used expectation maximization (Moon, 1996) to deal with these missing values.

### The Workshop

The design of the workshop was in line with recent job crafting interventions (e.g., Gordon et al., 2018; Dubbelt et al., 2019) and was tailored to meet the needs of the unemployment agency. The focus of the unemployment agency is on providing high quality services to their unemployed customers. Therefore, we extended the workshop with 90 min (bringing the total to 5.5 h) to train participants on how job crafting could help them to provide optimal services to their unemployed customers.

During the first day, the intervention focused on theory and practicing with job crafting (see Table 1A). Participants set four SMART goals (Doran, 1981) which they chose themselves and worked on them in the weeks between the first and second day. Participants received handouts of the presentation and a workbook in which they could take notes and formulate their job crafting goals. The first author, who is an experienced trainer, gave the workshops herself. Participants were told that the aims of the intervention were to increase job crafting behavior, work engagement and providing high quality services. Therefore, the workshop focused on employee’s needs, past (success) experiences, the current work situation and the desired work situation. To make sure the intervention covered the needs of employees within the unemployment agency, we conducted interviews (*N* = 19) with employees from the various departments prior to the intervention. Overall, the interviews showed that people felt the need for a manageable workload, clear targets and more performance feedback. Additionally, they expressed the need for more role clarity and better communication with management and between the different departments. During the training, these results were used to set examples and to inspire employees to see potential ways in which they could implement job crafting into their work. Six weeks after the intervention, participants discussed their job crafting experiences during an evaluation session (see Table 1B). Participants reflected upon their experiences and thought about ways to implement job crafting in their work routines beyond the intervention. In the weeks between the first and second training day, people received a weekly reminder to help them work on their job crafting goals. A week before the second training day a reminder was sent to invite people to participate in the upcoming session. All training sessions and additional contact (via email) was standardized. Checklists were available for the trainer to check whether everything was discussed. Moreover, a timetable was maintained during each training session. This standardization process was conducted in order to actively maintain intervention fidelity (van Zyl et al., 2019).

### Measures

#### Job Crafting

Job crafting was measured using the Job Crafting Scale (JCS) developed by Tims et al. (2012). The scale consists of 21 items, representing four sub dimensions: *increasing structural job resources* (5 items, e.g., I try to learn new things at work; α T1/T2 were, respectively, 0.79/0.83), *increasing social job resources* (5 items, e.g., I ask others for feedback on my performance; α T1/T2 were, respectively, 0.77/0.78), *decreasing hindering job demands* (6 items, e.g., I make sure my work is mentally less intense; α T1/T2 were, respectively, 0.76/0.77) and *increasing challenging job demands* (5 items, e.g., When an interesting projects comes along, I offer myself proactively as project co-worker; α T1/T2 were, respectively, 0.88/0.88). Items were measured on a 5-point Likert scale ranging from 1 = never to 5 = always. Cronbach’s alphas for the overall construct (T1/T2) were, respectively, 0.84/0.84.

#### Work Engagement

Work engagement was assessed using the short version of the Utrecht Work Engagement Scale (UWES) (Schaufeli et al., 2006). Nine items represent three sub dimensions: *vigor* (three items, e.g., At my work, I feel bursting with energy; α T1/T2 were, respectively, 0.84/0.85), *dedication* (3 items, e.g., I am enthusiastic about my job; α T1/T2 were, respectively, 0.91/0.89) and *absorption* (3 items, e.g., I am immersed in my work; α T1/T2 were, respectively, 0.79/0.75). Items were measured on a 7-point Likert scale, ranging from 0 = never to 6 = always. Cronbach’s alphas for the overall construct (T1/T2) were, respectively, 0.92/0.91.

#### Empowerment

Empowerment was measured using the 12-item scale developed by Spreitzer (1995). This scale consists of four sub dimensions covering *competence* (three items e.g., I am confident about my ability to do my job; α T1/T2 were, respectively, 0.90/0.93), *self-determination* (three items, e.g., I can decide on my own how to go about doing my job; α T1/T2 were, respectively, 0.92/0.88), *impact* (three items, e.g., My impact on what happens in my department is large; α T1/T2 were, respectively, 0.93/0.92) and *meaning* (three items, e.g., The work I do is meaningful to me; α T1/T2 were, respectively, 0.95/0.95). Items were measured on a 7-point Likert scale ranging from 1 = never to 7 = always. Cronbach’s alphas for the overall construct (T1/T2) were, respectively, 0.89/0.89.

#### Service-Oriented Task Performance^1^

Service-oriented task performance was assessed using the scale developed by ‘the authors’, (*submitted*). This scale consisted of five items: ‘I am meaningful for my customers,’ ‘I listen to the concerns my customers have,’ ‘I take the time to fulfill the emotional needs of my customers,’ ‘I give clear explanations about the rules of the unemployment agency’ and ‘I support my customers in their search for a new job’ (α T1/T2 were, respectively, 0.89/0.89). Items were measured on a 5-point Likert scale ranging from 1 = totally disagree to 5 = totally agree.

#### Empowering Service^1^

Empowering service was measured using an adapted version of the Empowering Leadership Scale (ELS) developed by Amundsen and Martinsen (2014). This scale was originally developed for managers and leaders, but is also relevant for the unemployment sector since service employees and their unemployed customers have a hierarchical relationship too. We included 18 items which were adapted to match a service providing environment. Example items were ‘I encourage my customers to take their own initiative when looking for a job’ and ‘I recognize the strong and weak sides of my customers’ (α T1/T2 were, respectively, 0.94/0.92). Items were measured on a 5-point Likert scale ranging from 1 = never to 5 = always.

#### Customer Ratings

Customer ratings were assessed using the 18 items from the empowering service scale, transcribed to the customer’s perspective (e.g., ‘My advisor encourages me to take personal initiative when looking for a job’ and ‘My advisor recognizes my strong and weak sides’). Customers rated the performance of their advisor (1 = completely disagree; 5 = completely agree). Cronbach’s alphas (T1/T2) were, respectively, 0.98/0.98.

### Strategy of Analysis

Data were analyzed using General Linear Modeling (GLM) repeated measures in SPSS to test the effects of the intervention over time. We conducted a two-way repeated measure analyses of variance with a time (T1 and T2 measure) by group (intervention and control) design. Time was used as the within-person factor and group as the between-person factor. Afterward, we conducted paired sample *t*-tests to explore differences within the groups. Customer satisfaction measures were analyzed using *t*-tests.

## Results

**FIGURE 3:**
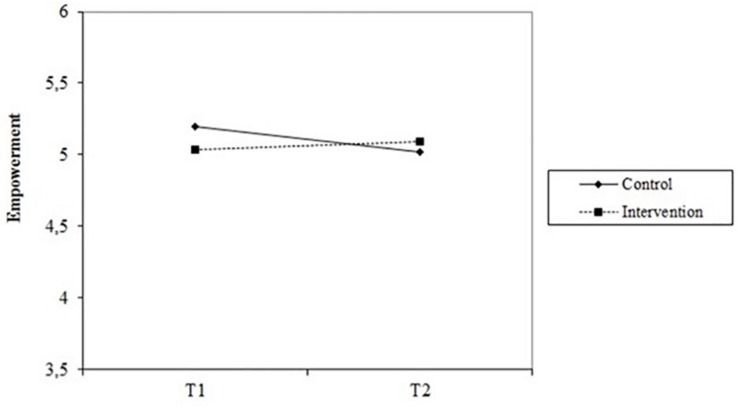
Interaction effect of group (intervention and control) ^∗^ time (T1 and T2) for employee empowerment.

Table 3 shows the intercorrelations between the study variables on T1 and T2. Table 4 shows the means, standard deviations, results of the repeated measures ANOVAs and *t*-tests and the effect sizes for the study variables. Hypothesis 1a–d examined whether employees in the intervention group showed higher levels of all types of job crafting behavior compared to the control group. Based on the repeated measures ANOVAs, H1a–H1d are rejected (*F*_increasing structural resources_ = 0.57, *p* = n.s.; *F*_increasing social resources_ = 0.16, *p* = n.s.; *F*_decreasing hindering demands_ = 2.61, *p* = n.s.; F_increasing challenging demands_ = 0.90, *p* = n.s.). However, when conducting t-tests to explore the growth patterns within both groups, results showed that employees in the intervention group experienced an increase in decreasing hindering demands (*t* = −2.76, *p* < 0.01) from T1 to T2, while employees in the control group did not (*t* = −0.16, *p* = n.s.). Thus, even though H1a–d are rejected based on the repeated measures ANOVAs, the *t*-test for decreasing hindering demands provides some support for hypothesis 1c. Given the Cohen’s d of 0.26 (95% CI [0.12 – 0.39]), this effect is small at best. Hypothesis 2a-b examined whether employees in the control group experienced a decrease in work engagement and empowerment compared to the intervention group. Based on the repeated measures ANOVAs H2a is rejected (*F*_work engagement_ = 0.66, *p* = n.s.). H2b is accepted (*F*_empowerment_ = 4.33, *p* = 0.04), as empowerment in the control group is declining (*t* = 2.49, *p* = 0.02) from T1 to T2, while in the experimental group it is not (*t* = −0.56, *p* = n.s.), see Figure 3. Given the Cohen’s *d* in the control group of −0.26 (95% CI [−0.42 – −0.12]), the effect of the decreasing levels of empowerment is small. When conducting t-tests to compare growth patterns within each group for H2a, results showed that employees in the control group experienced a decrease in work engagement (*t* = 2.04, *p* = 0.05) from T1 to T2, while employees in the intervention group did not (*t* = 1.18, *p* = n.s.). With a Cohen’s *d* of −0.20 (95% CI [−0.43 – −0.01]) for the control group, the overall effect of loss of work engagement is small. Overall, H2b is accepted while H2a is rejected, although the *t-*test provide some support for a buffering effect (i.e., no decrease) in work engagement for the intervention group. Hypothesis 3a-b examined if employees participating in the job crafting intervention were able to maintain their levels of service-oriented task performance and empowering service, while employees in the control group could not. Based on the repeated measures ANOVAs and *t*-tests, both H3a and H3b are rejected as both employees in the control group and the intervention group were able to maintain their (self-rated) levels of performance (*F*_service–oriented task performance_ = 1.50, *p* = n.s., *t*_sevice–oriented task performance_ = 0.61, *p* = n.s.; *F*_empowering service_ = 0.004, *p* = n.s., *t*_empowering service_ = −0.41, *p* = n.s.). Hypothesis 4 examined whether customer ratings were higher for employees in the intervention group compared to employees in the control group before the start of the intervention, 5 months later and 1 year later. We used a *t-*test^2^ to explore the differences between the groups and found no differences between the intervention group and control group before the start of the intervention (*t* = 0.49, *df* = 94, *p* = n.s.) and 5 months after the intervention (*t* = −0.21, *df* = 101, *p* = n.s.). Therefore, hypothesis 4a is rejected. We did find an effect on customer ratings of empowering service 1 year after the intervention, as employees in the control group were rated more positively than their peers in the control group (*t* = −2,51, *df* = 179, *p* = 0.01). Therefore, hypothesis 4b is accepted.

**TABLE 3:** Intercorrelations between the study variables for the pre- (T1) and post- (T2) measure (*N*_T__1_ = 163; *N*_T__2_ = 127).

Variable	1	2	3	4	5	6	7	8
(1) Increasing structural resources		–0.02	0.29**	0.54**	0.55**	0.50**	0.28**	0.38**
(2) Decreasing hindering demands	–0.04		0.31**	0.02	0.06	<0.01	–0.08	< −0.01
(3) Increasing social resources	0.41**	0.22**		0.30**	0.33**	0.18^∗^	–0.05	0.11
(4) Increasing challenging demands	0.59**	–0.02	0.38**		0.42**	0.35**	0.17	0.31**
(5) Work engagement	0.53**	0.08	0.29**	0.37**		0.66**	0.14	0.38**
(6) Empowerment	0.35**	0.03	0.06	0.17*	0.56**		0.35**	0.31**
(7) Service-oriented task performance	0.36**	–0.06	0.06	0.23*	0.32**	0.33**		0.45**
(8) Empowering service	0.32**	−0.21*	0.12	0.25**	0.30**	0.21*	0.57**	

Correlations within the pre-(T1) measure are shown under the diagonal, correlations within the post-(T2) measure are shown above the diagonal. *p < 0.05; **p < 0.01.

**TABLE 4:** Mean, SD, *t*-test, effect size and repeated measures ANOVA’s for the study variables.

	Experimental group (*N* = 66)						Control group (*N* = 61)						RM ANOVA		
Variable	*M*	*SD*	*t*-Test *t*	*p*^a^	Cohen’s *d*	95% CI	*M*	*SD*	*t*-Test *t*	*p*^b^	Cohen’s *d*	95% CI	*F*	*p*^c^	Cohen’s *d*
JC: ISTR (T1)	3.99	0.52	−0.12	0.90	0.02	[−0.12 – 0.14]	3.81	0.48	−1.32	0.19	0.13	[0.01 – 0.23]	0.57	0.45	0.14
JC: ISTR (T2)	4.00	0.55					3.87	0.47							
JC: DHD (T1)	2.42	0.53	−2.76	0.008**	0.26	[0.12 – 0.39]	2.44	0.58	−0.16	0.87	0.03	[−0.11 – 0.15]	2.61	0.11	0.29
JC: DHD (T2)	2.57	0.61					2.46	0.59							
JC: ISOR (T1)	2.70	0.49	−0.44	0.66	0.04	[−0.08 – 0.15]	2.51	0.64	−0.85	0.40	0.03	[−0.12 – 0.17]	0.16	0.69	0.07
JC: ISOR (T2)	2.72	0.50					2.53	0.60							
JC: ICD (T1)	3.51	0.75	−0.44	0.66	0.03	[−0.15 – 0.20]	3.16	0.74	0.87	0.39	−0.07	[−0.26 – 0.06]	0.90	0.35	0.17
JC: ICD (T2)	3.53	0.73					3.11	0.72							
WE (T1)	4.83	1.01	1.18	0.24	−0.09	[−0.32 – 0.14]	4.92	0.91	2.04	0.05*	−0.20	[−0.43 – −0.01]	0.60	0.44	0.12
WE (T2)	4.74	0.93					4.74	0.94							
EMP (T1)	5.07	0.74	−0.56	0.58	0.06	[−0.12 – 0.22]	5.23	0.67	2.49	0.02*	−0.26	[−0.42 – −0.12]	4.33	0.04*	0.37
EMP (T2)	5.11	0.71					5.06	0.66							
S-OTP (T1)^d^	4.55	0.36	0.61	0.55^da^	−0.08	[−0.19 – 0.02]	4.37	0.73	−1.11	0.28^db^	0.10	[−0.14 – 0.35]	1.50	0.22^dc^	0.29
S-OTP (T2)^d^	4.52	0.38					4.44	0.69							
EMP SE (T1)^d^	4.33	0.40	−0.41	0.69^da^	0.05	[−0.06 – 0.17]	4.27	0.45	−0.45	0.66^db^	0.07	[−0.06 – 0.23]	0.004	0.95^dc^	0.02
EMP SE (T2)^d^	4.35	0.37					4.30	0.39							

JC, job crafting; ISTR, increasing structural resources; DHD, reducing hindering demands; ISOR, increasing social resources; ICD, increasing challenging demands; WE, work engagement; EMP, empowerment; S-OTP, Service-oriented task performance and EMP SE, empowering service. Repeated measures ANOVA: Time (within-person) × Group (between-person). *^*a*^*df = 65; ^*b*^df = 60; ^*c*^df = 1, 125. ^*d*^*N*_*experimental group*_ = 44; *N*_*control group*_ = 33. ^*da*^df = 43; ^*db*^df = 32; ^*dc*^df = 1, 75. *p ≤ 0.05; **p < 0.01.

## Discussion

In this study, we examined the effects of a job crafting intervention, in which employees of a Dutch unemployment agency learned to implement job crafting behaviors in their work routines. The intervention aimed to prevent a decrease in empowerment, work engagement and employee performance (i.e., the provision of high-quality services) due to organizational changes. During a 1-day intervention, employees learned about job crafting and how to implement it into their work. Six weeks after the first day, they came back for a reflection session, in which they evaluated the effects of job crafting on their work. Both days were based on experiential learning theory (Kolb et al., 2000).

Our intervention increased one of the four job crafting behaviors (i.e., decreasing hindering demands). Furthermore, results showed that the intervention was successful to prevent a decrease in employee empowerment and work engagement. We did not find an effect for service-oriented task performance and empowering service as both the intervention group and the control group were able to maintain their performance levels. However, the effects of empowering service were noticeable for customers, as 1 year after the intervention customers were more positive about the service quality of employees in the intervention group compared to the control group. Overall, our results showed that the intervention may be a promising tool to maintain employee well-being during times of change. However, as will be discussed in more detail below, not all results were in line with our expectations.

First, we found support that the intervention had an effect on aspects of employee well-being. Using RM ANOVA’s, our results showed that employees in the control group experienced a decrease in empowerment, whereas employees in the intervention group did not. Employee empowerment in the intervention group was stable over time, regardless of any changes going on in the organization. Furthermore, we found some preliminary support that the intervention had an effect on work engagement, as employees in the control group showed a decrease in work engagement, whereas employees in the intervention group did not. However, these effects were detected using *t*-tests (which considers pre- and post-measures within the same group) but were not supported by the RM ANOVA (which considers both the intervention and control group simultaneously). Therefore, these results must be interpreted with caution. Nevertheless, they seem promising for the future. Based on our findings, we conclude that the job crafting intervention holds the potential to sustain employee well-being (i.e., empowerment and possibly work engagement). It provides employees with additional (personal) resources to adapt to increasing demands in an efficient way. When employees craft their job, they are suggested to enhance the fit between themselves and their work (Bakker et al., 2012). This is extremely valuable, especially during times of organizational change, as it provides employees with a sense of control and self-efficacy. Moreover, it helps to prevent feeling of powerlessness as a consequence of the organizational changes (Conger and Kanungo, 1988; Van Den Heuvel et al., 2013). Furthermore, through job crafting, employees can maintain their work engagement. This is not only valuable for the employee him-/herself, but also for the organization. Engaged employees like their job, feel energized while working and have a lower risk of stress-related health issues like burn-out (Bakker et al., 2014). Moreover, as work engagement is contagious (Bakker and Demerouti, 2009) employees engaged in their job can act as a counterforce toward any possible cynicism regarding the organizational changes going on (Van den Heuvel et al., 2010). Moreover, such employees are more creative and willing to go the extra mile. This is important, as organizational change is hardly ever a smooth process (Cartwright and Holmes, 2006; Kompier, 2006). Thus, to protect employee well-being during times of change, the job crafting intervention is a valuable tool as it helps employees to adapt to the heightened demands.

The intervention had, unexpectedly, little effect on reported job crafting behavior. Results did show a significant increase in decreasing hindering demands for the intervention group, when conducting *t*-tests. Thus, employees in the intervention group showed an increase in decreasing hindering demands when comparing their pre- and post-measure. Although this result must be interpreted with caution, it is in line with the results of Oprea et al.’s (2019) meta-analysis which also showed that job crafting interventions have a stronger effect on the reducing demands dimension. One reason that the intervention seemed to have only little effect on reported job crafting behavior may be that employees specified very specific crafting goals to optimize their working conditions, which may not be captured by the (more generally formulated) items used to measure job crafting behavior (Van den Heuvel et al., 2015). For example, one participant worked in an open office space where it was hard to focus as people were making phone calls all day. Therefore, the participant decided to wear earplugs when carrying out tasks that needed full concentration. As researchers, we would classify this as reducing hindering demands. However, this specific behavior may not be captured by the items for reducing demands (e.g., ‘I make sure my work is mentally less intense’). Although we used a more specific measure of job crafting in line with the suggestions of Van den Heuvel et al. (2015), it may be difficult for participants to see the link between their actual behavior and the job crafting items. One suggestion to improve the intervention is to gather and document the different job crafting goals set and check (at T2) whether participants worked (successfully) on them. This way, it is possible to overcome the discrepancy between the job crafting items and the actual job crafting behaviors. Another reason that the intervention seemingly had little effect on job crafting behaviors, may be the organizational changes going on, especially when it comes to increasing challenging demands and seeking (structural and social) resources. Both structural and social resources may have been less available. Work load was increasing, as employees had to see twice as many clients. This may have inhibited them to provide resources to each other as they had other priorities. Furthermore, structural resources may have been harder to get as the organization required their employees to do more with less means. As for increasing challenging demands: the new targets asked a lot of extra effort form employees and they may be considered a (huge) challenge in itself. Therefore, as employees had to adjust to the changes, they may not have had sufficient time (or even motivation) to seek additional challenges.

The intervention had no effect on (self-reported) service quality. We found no differences between the intervention and the control group for both service-oriented task performance and empowering service, although we expected the control group to decline. One possible explanation that the control group did not show a decline may be that the timeframe between the two measurement points was too short. Employees experiencing high job demands over a prolonged period of time may become chronically exhausted, and this may negatively affect their performance (Bakker and Demerouti, 2017). We used a timeframe of 6 weeks and this may have been too short to be labeled as ‘prolonged exposure.’ Another reason that we did not find any differences in performance, might be that people tend to be more lenient toward their own performance (Holzbach, 1978). Therefore, we have also taken customer-rated measures of performance into account and found that 1 year after the intervention, employees in the intervention group were rated significantly more positive by their customers than employees in the control group. However, this effect was not found 5 months after the intervention. A possible explanation for finding differences in customer-rated measures of performance 1 year after the intervention, but not yet after 5 months, may be that behavioral change takes time. Lally et al. (2010) showed that on average it took 66 days to develop daily routines, but the range of habit formation was widespread, from 18 up to 254 days. In their study they examined the formation of simple (health-related) habits like drinking a bottle of water during lunch. However, the provision of high-quality services is much more complex and thus it may take longer for employees to change their behavior and form new habits. Moreover, Lally et al. (2010) state that habit formation is easier in a stable context. As organizational changes were implemented and as each client requires a different approach, the context was anything but stable, making it even harder for employees to change their behavior. Although we measured after 5 months, it may have been too early to notice any differences as the behavioral change was complex and the context was unstable. Nevertheless, our findings emphasize the importance of providing employees with sufficient tools (i.e., job crafting techniques) and resources to craft their work environment (Wrzesniewski and Dutton, 2001; Tims and Bakker, 2010).

Overall, our results are in line with and complementary to other studies regarding the job crafting intervention (e.g., Van den Heuvel et al., 2015; Demerouti et al., 2017; Gordon et al., 2018; Dubbelt et al., 2019) as it has shown to have an effect on employee well-being (i.e., empowerment and work engagement) as well as on service quality. Since the effects of the intervention on job crafting behavior were rather weak, the question that arises is how the intervention improved the outcomes. Demerouti et al.’s (2017) intervention that was conducted in an organizational change context showed that the effect of the intervention on openness to change and adaptive performance was explained by positive affect, instead of job crafting. Such an explanation could also be applicable in our study and is in line with the proposition of Oldham and Hackman (2010) that the benefits of job crafting may “derive from substantive changes in the work itself” (due to crafting) or “merely from having the opportunity to tailor one’s own work responsibilities” (p. 471).

### Practical Implications

This study is of practical relevance too. Many organizations are dealing with changes these days. They have to re-invent themselves to work more efficiently and they have to come up with innovative products and services to keep their customers satisfied (Chiang and Jang, 2008). As the pace in which changes are implemented is high (Kompier, 2006), employees have to adapt to these changes at a high pace too. Therefore, it is of vital importance for employees to have sufficient strategies to adapt to these changes. Moreover, to keep customers satisfied, it is important that employees provide optimal services and that these services are valued by their customers. Our study shows that through a job crafting intervention, employee well-being can be preserved during times of change, and that customer satisfaction can be enhanced, as 1 year after the job crafting intervention service employees in the intervention group are rated more positively by their customers than service employees in the control group. Therefore, as organizational changes are implemented, it is important for management to provide employees with sufficient tools (e.g., a job crafting intervention) to adequately adapt to and deal with the organizational changes going on. This way, employee well-being can be maintained, while customer satisfaction can even improve.

### Limitations and Future Research

When interpreting the results of this study, some limitations should be kept in mind. First, almost all variables were measured using self-reports, which can result in common method bias (Podsakoff et al., 2003). We used self-reports in line with the recommendations of Conway and Lance (2010) who state that self-reports should be used when trying to capture private experiences (e.g., work engagement, empowerment). Although we included customer ratings of service quality, future studies could try to incorporate more other-rated measures. For example, one could include peer ratings of job crafting behaviors and service-oriented task performance as both are behavioral measures and thus, are visible for others.

Second, we did not have a completely randomized study design – a limitation ever-present in field studies as organizations cannot force employees to participate. We tried to encourage participation by giving presentations in work meetings and by posting small messages in the organization’s weekly newsletter. However, we cannot rule out that only participants who were willing to change participated in the intervention. Nevertheless, employees in the intervention group did not differ from the control group on their pre-intervention scores, nor did they differ in age, tenure and years in their current position.

Third, although the intervention aimed to increase job crafting behavior, only one of the four job crafting dimensions significantly changed during the intervention period. As the intervention was delivered as intended, we cannot rule out that other factors, beside job crafting, influenced the results and being (at least partly) responsible for the effects found on work engagement, empowerment and performance.

Fourth, due to organizational constraints, it was impossible to collect customer satisfaction measures from the same customer over time. Therefore, we were forced to rely on t-test instead of RM-ANOVA’s when examining customer ratings. Future research including customer ratings should try to incorporate a follow-up over time to explore whether there are improvements in service using more advanced methods of analysis.

Lastly, in this study we used a single pre-intervention measure. Multiple pre-intervention measures might have helped us to determine the baseline more accurately, as the mere presence of researchers and the completion of questionnaires might already affect participants. Therefore, we encourage future researchers to use multiple pre-intervention measures to overcome this limitation.

Future research should examine the effectiveness of job crafting interventions in other contexts as well. We were the first to explore the job crafting intervention in a service setting during times of change. However, our results are mixed and not always in line with expectations. This highlights the importance of tailor-made interventions (Harden et al., 1999), designed to meet the requirements of different organizational phases (Swerissen and Crisp, 2004). For example, as increasing challenging demands might not be the most effective strategy during times of change, it may be beneficial for employees during more quiet times, as it can help to prevent boredom. More in-depth knowledge of the effects of job crafting interventions in various settings is extremely valuable, as only then interventions can effectively be tailored to adequately meet the specific needs of employees and the organization.

## Conclusion

This is the first study to explore the effects of a job crafting intervention in a service setting during times of organizational change. Our results highlight the importance of a job crafting intervention as a means to sustain empowerment among service employees. Empowerment is a crucial asset during times of change as empowered employees feel more confident and in control when carrying out their (changing) work, optimizing their performance (Erstad, 1997). This is also supported by our results, as employee performance – as rated by customers – was more positive up to 1 year after the intervention. These findings emphasize the importance of tailor-made job crafting interventions that provide employees with sufficient strategies to stay in control and preserve their level of performance during times of change.

## Data Availability Statement

The datasets generated for this study are available on request to the corresponding author.

## Ethics Statement

The studies involving human participants were reviewed and approved by the Onderzoekscommissie Industrial Engineering (OZC-IE). Written informed consent for participation was not required for this study in accordance with the national legislation and the institutional requirements.

## Author Contributions

All authors have made a substantial contribution to the conception of the manuscript, and the analysis and interpretation of the data. Moreover, all authors have been involved in the writing process.

## Conflict of Interest

The authors declare that the research was conducted in the absence of any commercial or financial relationships that could be construed as a potential conflict of interest.

## Footnotes

1 Not all participants in the intervention worked directly with customers. Therefore, these employees were excluded from the measure of service-oriented task performance and empowering service, resulting in *N*_intervention group_ = 44; *N*_control group_ = 33.

2 We were unable to conduct RM ANOVA’s for the customer satisfaction measures because of organizational reasons. Data were collected at three fixed points in time and it appeared impossible to schedule all the customers from T1 for an appointment with their advisor at T2 and T3 for the follow up.
